# Influence of Pre-Treatment and Artificial Aging on the Retention of 3D-Printed Permanent Composite Crowns

**DOI:** 10.3390/biomedicines10092186

**Published:** 2022-09-04

**Authors:** Tobias Graf, Kurt-Jürgen Erdelt, Jan-Frederik Güth, Daniel Edelhoff, Oliver Schubert, Josef Schweiger

**Affiliations:** 1 Department of Prosthetic Dentistry, Center for Dentistry and Oral Health, Goethe University Frankfurt, 60596 Frankfurt am Main, Germany; 2 Department of Prosthetic Dentistry, University Hospital, LMU Munich, 80336 Munich, Germany

**Keywords:** CAD-CAM, 3D printing, polymer-infiltrated ceramic network (PICN), resin nano ceramic (RNC), composite, permanent crowns, pull-off test, adhesive bonding

## Abstract

The aim of this in vitro study is to investigate the bonding properties of a 3D-printable permanent composite material in comparison to milled composite materials. The tested materials are 3D-printed BEGO VarseoSmile Crown ^plus^ (VA1_ab, VA1_nt, VA2_ab, VA2_nt), Vita Enamic (EN1, EN2), and 3M Lava Ultimate (UL1, UL2) (N *=* 64; n *=* 8). For this purpose, all crowns are luted to polymer tooth stumps #46 (FDI) using dual-curing luting composite, strictly according to the manufacturer’s instructions. VA1_ab and VA2_ab are additionally airborne-particle abraded. 4 groups (VA2_ab, VA2_nt, EN2, UL2) are artificially aged (1,200,000 cycles, 50 N, 10,000 thermocycles), whereby no specimen has failed. All 64 specimens undergo pull-off testing until retention loss. The mean forces of retention-loss is 786.6 ± 137.6 N (VA1_nt, *), 988.6 ± 212.1 N (VA2_nt, *, Ɨ), 1223.8 ± 119.2 N (VA1_ab, Ɨ, ǂ), 1051.9 ± 107.2 N (VA2_ab, *, Ɨ), 1185.9 ± 211.8 N (EN1, Ɨ, ǂ), 1485.0 ± 198.2 N EN2, ǂ), 1533.8 ± 42.4 N (UL1, ǂ), and 1521.8 ± 343.4 N (UL2, ǂ) (one-way ANOVA (Scheffé method); *p* < 0.05; *, Ɨ, ǂ: group distribution). No characteristic failure modes can be detected. In conclusion, all of the pull-off forces reflect retention values that seem to be sufficiently high for clinical use. Additional airborne-particle abrasion of VA does not result in significantly better retention but can be recommended.

## 1. Introduction

Three-dimensional (3D)-printing processes are used in many areas of dental technology and dentistry. Whereas CAD-CAM technology led to a paradigm shift for new materials and treatment concepts already in the mid-2000s, recently ever more 3D-printed applications emerge, such as 3D-printed surgical templates, individual impression trays, models, occlusal splints, or metal frameworks [1,2,3]. A key advantage is the efficiency of the manufacturing process, reducing the waste of material compared to subtractive milling processes [1]. Moreover, mechanical and aesthetic characteristics of the 3D workpiece can be tailored specifically to individual esthetic requirements by adapting the composition of the substrate, e.g., by the addition of color particles [2].

In February 2020, a material (VarseoSmile Crown ^plus^; BEGO, Bremen, Germany; here: VA) for additive manufacturing of tooth-colored definitive dental restorations became available. It is approved for single-tooth restorations such as full crowns, inlays, onlays, or veneers, and has been—to the knowledge of the authors—the first available 3D-printable material approved for definitive dental restorations [4]. The material is a ceramic-infiltrated hybrid composite consisting of a methacrylic ester matrix with ceramic fillers and can be classified as a “resin matrix ceramic” (RMCs) [5].

Evidence-based clinical data on VA are not yet available. When considering the clinical behavior of other millable RMCs, clinical data are currently available for a maximum of four years [6,7,8,9]. Fathy et al. reported success rates from 93.5 to 100% in their recent systematic review, which contains data between one and three years. However, data beyond this period are still lacking [8]. Debondings and fractures are the most prevalent reported complications [8,9]. Over 50% of all decementations occur within a short period—namely the first six months of clinical use. According to Kabetani et al., the taper of the preparation design is more critical in order to prevent complications than the vertical dimension of the abutment teeth or the thickness of the occlusal surface of the crown [9]. Nevertheless, material properties such as a sufficiently high flexural strength and Young’s modulus as well as an adequate adhesive bonding to the tooth stump are also essential parameters for mid- and long-term success in regards to retention loss.

Since RMCs have a flexural strength of significantly less than 350 MPa [10], a fully adhesive luting procedure is recommended for RMC restorations to enable an optimum connection between tooth and restoration [11]. This results in a stable tooth-restoration complex which cannot be achieved by conventional cementation. In various studies, the restorations were successfully attached using different resin cements, such as ResiCem (SHOFU DENTAL GmbH, Ratingen, Germany), Clearfil SA luting (Kuraray Europe GmbH, Hattersheim, Germany), Panavia F2.0 (Kuraray Europe GmbH, Hattersheim, Germany), or Panavia V5 (Kuraray Europe GmbH, Hattersheim, Germany) [8,9]. Furthermore, appropriate moisture control and strict adherence to the manufacturers’ instructions during the insertion procedure contribute significantly to success [12].

Some RMCs should be airborne-particle abraded before placement to generate an optimum bond strength [13], for example, Lava Ultimate (resin nano ceramic; 3M Deutschland GmbH, Neuss, Germany) (UL) according to their manufacturer’s recommendations. The resin infiltrated ceramic (VITA Enamic; Vita Zahnfabrik, Bad Säckingen, Germany, Germany) (EN), on the other hand, needs to be etched with hydrofluoric acid before luting [12,14]. For VA, neither airborne-particle abrasion nor etching of the restoration is recommended before bonding [4]. Contrary to glass ceramics, etching with hydrofluoric acid cannot dissolve out the amorphous glass matrix in VA, and is hence pointless. However, airborne-particle abrasion of VA restorations may perhaps lead to increased bond strength values and might therefore minimize the risk of clinical retention loss.

The present study aims to investigate whether the novel 3D-printable material VA shows pull-off resistance comparable to other RMCs that have been available on the dental market for a longer period of time. The null hypotheses are that VarseoSmile Crown ^plus^ (VA) presents no significant differences regarding pull-off forces compared to Enamic (EN) or Lava Ultimate (UL) and that an additional pretreatment step of airborne-particle abrasion has no significant influence on the pull-off forces.

## 2. Material and Method

### 2.1. Fabrication of Test Specimens

**Figure 1:**
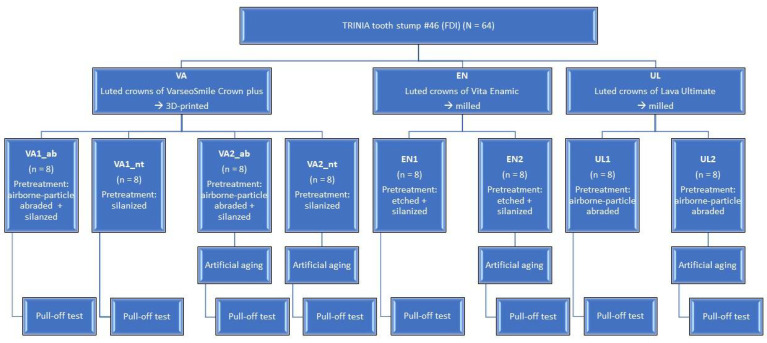
Experimental setup.

A total of 64 test specimens distributed to eight test groups were prepared for the study (N *=* 64) and three different crown materials were examined. The number of specimens per test group was n *=* 8 (Figure 1 and Table 1).

**Figure 2:**
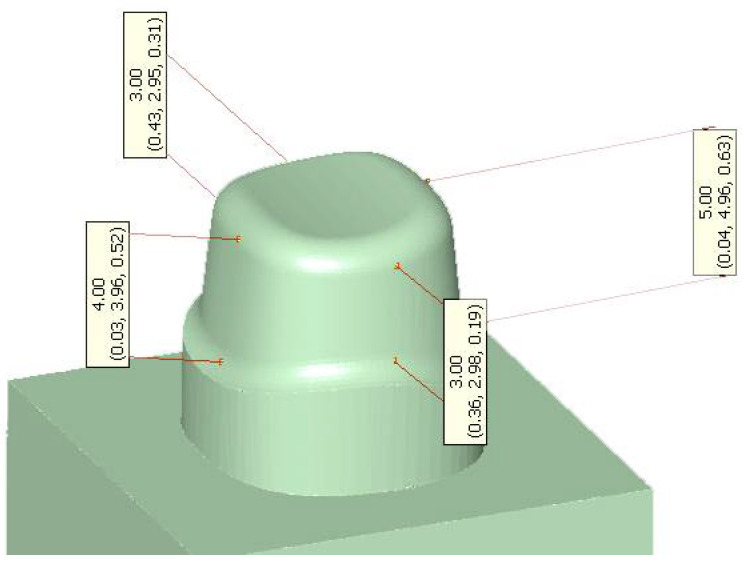
Design of the CAD-CAM milled tooth stump and its baseplate (FDI #46).

A tooth stump (FDI #46) was designed with a baseplate using CAD-Software “Solid Works 3D CAD Version 2019“ (Dassault Systèmes, Vélizy-Villacoublay, France). The preparation angle was 12° (α/2 < 6°). The lateral height of the prepared stump was 3.0 mm mesial and distal, 5.0 mm buccal, and 4.0 mm lingual (Figure 2). The resulting data set was milled at BEGO Medical (Bremen, Germany) from TRINIA blanks (Bicon, Boston, MA, USA); 64 identical tooth stumps formed the basis for all crowns to be bonded.

**Figure 3:**
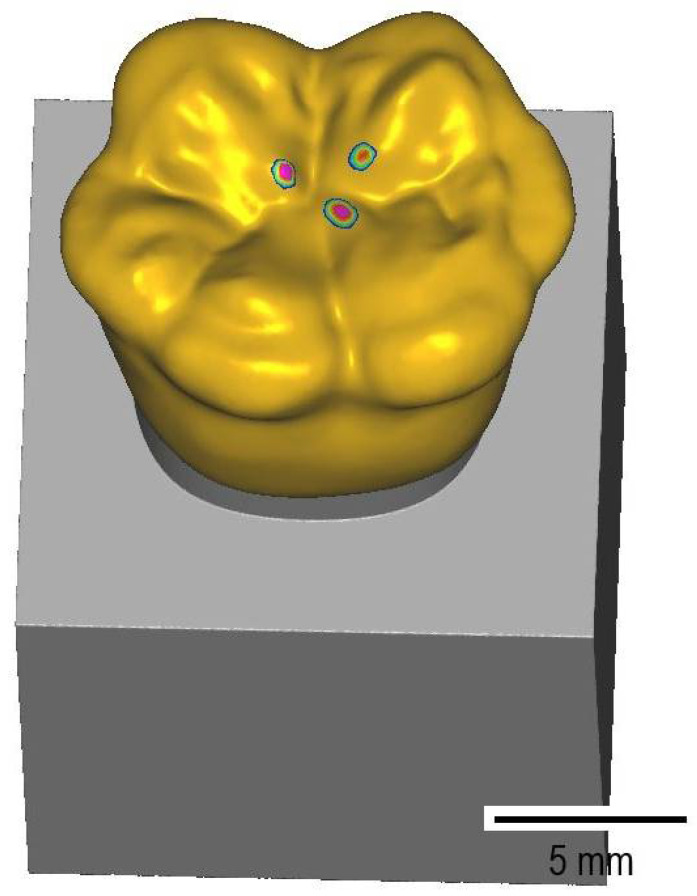
STL-file of the crown with retention geometries and a generated three-point contact in the central fissure for the antagonist metal stylus.

Single-tooth crowns made from three materials, i.e., VarseoSmile Crown ^plus^ (VA; BEGO, Bremen, Germany), Lava Ultimate (UL; 3M GmbH, Neuss, Germany), and Enamic (EN; Vita Zahnfabrik, Bad Säckingen, Germany), were fabricated. The CAD design of the crowns was carried out (Modelier CAD-Software v.6173_6843_x64; Zirkonzahn GmbH, Gais, Italy) on the basis of a molar from an existing database. A thickness of 1.0 mm was set as the minimum occlusal and circumferential wall thickness. In order to allow efficient fixation of the crown in the extractor device, a free-form tool was used to design retention geometries in the lateral areas of the occlusal surface. In preparation for the artificial aging process, the STL file of the antagonistic metal stylus was imported into the software and a three-point contact was generated in the central fissure of the occlusal surface (Figure 3).

The crowns of VA were produced using the “digital light processing” (DLP) 3D-printer Varseo XS (wavelength: 405 nm, resolution (X, Y, Z): 50 µm; building speed: 30 mm/h; BEGO, Bremen, Germany) following strictly the manufacturer’s recommendation. After 3D printing, all crowns of VA were cleaned by an ultrasonic unit with ethanol for 5 min and airborne-particle abraded with Perlablast (50 µm; BEGO, Bremen, Germany) at 1.5 bar. Then, after the polishing procedure, they were exposed in a light-curing device BEGO Otoflash (2 × 1500 flashes in N_2_ atmosphere; BEGO, Bremen, Germany) for post-curing. The crowns of groups EN and UL were fabricated by using the CNC milling machine imes Icore 350i (imes-icore GmbH, Eiterfeld, Germany).

### 2.2. Adhesive Luting Protocols

As part of the bonding process, all TRINIA tooth stumps were airborne-particle abraded with aluminum oxide (grit size 110 μm at 1.5 bar), Scotchbond Universal adhesive (3M Deutschland GmbH, Neuss) was applied, and light cured for 10 s with LED polymerization light Bluephase Style (Ivoclar Vivadent AG, Schaan, Liechtenstein, Liechtenstein).

**Figure 4:**
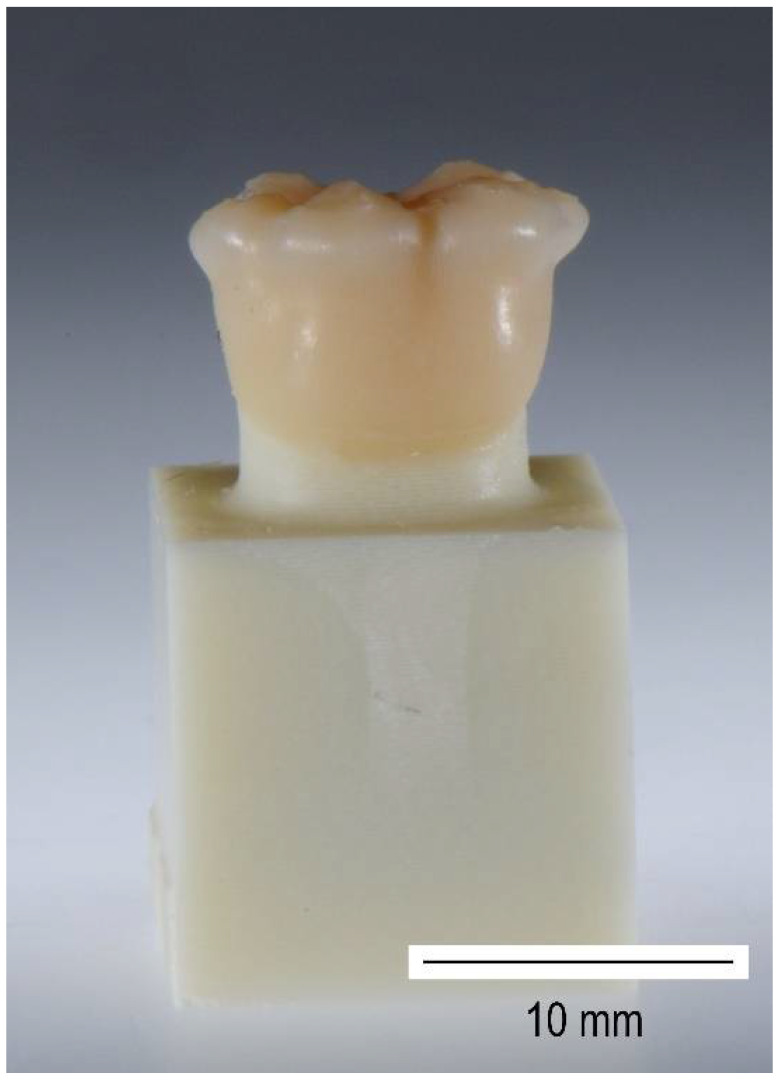
Exemplary manufactured specimen of VA1_nt.

The pretreatment of the crowns varied: The inner surfaces of the crowns of VA1_ab and VA2_ab were airborne-particle abraded with Korox (Al2O3, 50 μm, 1.5 bar, distance 5 cm) (BEGO, Bremen, Germany), whereas this procedure was not performed for VA1_nt and VA2_nt. All VA-crowns were cleaned with Ivoclean (Ivoclar Vivadent AG, Schaan, Liechtenstein) and then Monobond Plus (Ivoclar Vivadent AG, Schaan, Liechtenstein) was applied to the bonding surface (Figure 4).

EN was etched on the bonding surfaces for 60 s using 5% hydrofluoric acid gel (VITA ADIVA CERA-ETCH 5%; Vita Zahnfabrik, Bad Säckingen, Germany) before VITA ADIVA C-PRIME (silane bonding agent; Vita Zahnfabrik, Bad Säckingen, Germany) was applied to the etched surface. UL was airborne-particle abraded with Korox (Al_2_O_3_, 50 μm, 1.5 bar, distance 5 cm) (BEGO, Bremen, Germany), then brushed with Scotchbond Universal adhesive (3M Deutschland GmbH, Neuss, Neuss), but not light cured.

Variolink Esthetic DC (Ivoclar Vivadent AG, Schaan, Liechtenstein) was used for luting VA and EN, and RelyX Ultimate (3M Deutschland GmbH, Neuss) for UL. Tooth stump and restoration were mounted into a special cementation device and an axial load of 30N was applied to the complex with a stylus during the light curing process. The restoration margins were covered with a Liquid Strip (Ivoclar Vivadent AG, Schaan, Liechtenstein) after excess removal, whereupon the test specimens were cured for a total of 1:00 min using LED polymerization light Bluephase Style (Ivoclar Vivadent AG, Schaan, Liechtenstein). The bonding processes followed exactly the manufacturers’ instructions.

### 2.3. Artificial Aging and Pull-Off Test

Thirty-two of the sixty-four specimens, i.e., VA2_ab, VA2_nt, EN2, as well as UL2, were artificially aged at 50 N loading with 1,200,000 cycles (1.2 Hz) and 10,000 thermocycles (5 °C/ 55 °C; 30 s dwelling time of each temperature) (CS-4 chewing simulator; SD Mechatronik, Feldkirchen-Westerham, Germany). The remaining specimens (VA1_ab, VA1_nt, EN1, UL1) underwent pull-off testing without aging.

**Figure 5:**
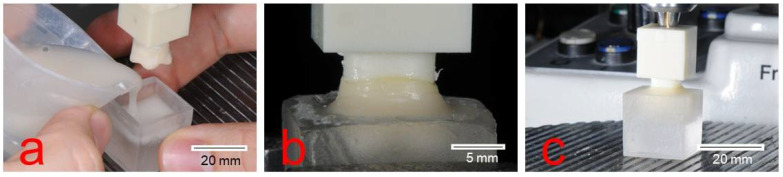
Embedding procedure of the test specimens for the pull-off test (**a**–**c**).

**Figure 6:**
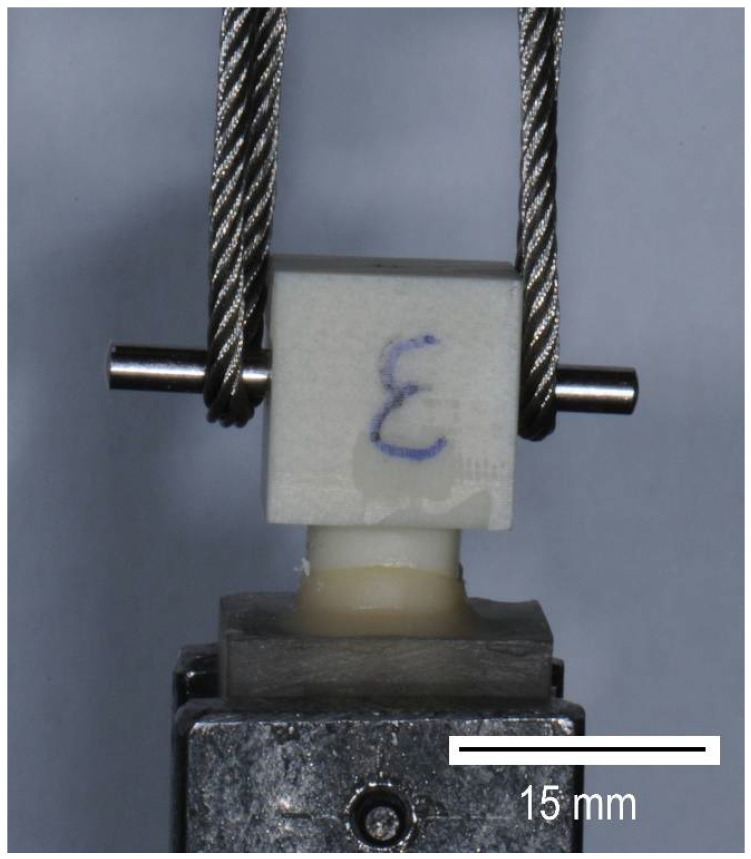
Pull-off device “Type 1445 universal testing machine” (Zwick GmbH & Co. KG, Ulm, Germany) including a mounted test specimen.

Before the pull-off testing, all crowns were embedded into resin blocks (Paladur, Kulzer GmbH, Hanau, Germany). Curing was performed without any application of pressure or temperature to avoid influencing the bonding connection between the crown and stump (Figure 5). Central transverse holes were drilled into both, stump base and crown base. Metal bolts, which were used for fixing the test specimens during the pull-off device, were mounted through these holes. The specimens were fixed into the testing machine using a flexible steel cable (Figure 6) to prevent shear loading.

The pull-off tests were performed with a type 1445 universal testing system (Zwick GmbH & Co. KG, Ulm, Germany). The software (testXpert, 7.11/d, Zwick GmbH & Co. KG, Ulm, Germany) allowed the speed- and position-control of a mobile traverse. The crown pull-off tests were conducted at a test speed of 0.5 mm/min and a preliminary test force of 0 N. The force cut-off threshold was set at 80% F_max_. The results of the measurements were recorded in force-path diagrams.

**Figure 7:**
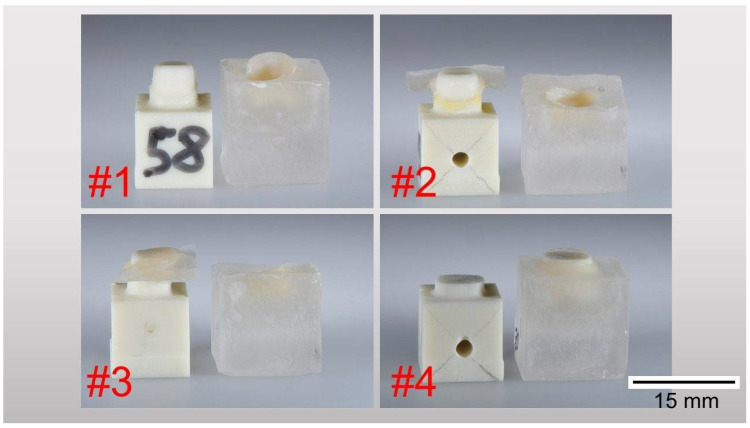
Illustration of the defined failure criteria: #1 “Complete debonding”, #2 “Partial debonding”, #3 “ Debonding of the occlusal crown surface”, #4 “Stump fractured”.

After testing, specimens were inspected to identify and classify the debonding behavior. Four categories were defined (Figure 7):

- #1: “Complete debonding” (more than 50% of the lateral stump surface detached from the crown),
- #2: “Partial debonding” (less than 50% of the lateral stump surface detached from the crown),
- #3: “Debonding of the occlusal crown surface” (lateral crown surface adheres to the stump—excluded the occlusal surface), and
- #4: “Stump fractured”.

### 2.4. Statistics

The data gathered concerning the crown pull-off forces were imported into a statistic program (Statistics 23.0, SPSS Inc., Stanford, CA, USA) for statistical processing, prepared for analysis, and subsequently evaluated. The Kolmogorov–Smirnov test was used to verify the normal distribution of the values within the test groups. All test groups were analyzed in a one-way analysis of variance (ANOVA). Differences between the groups were examined by the Scheffè method post hoc test. The level of significance was set at 5% (*p* < 0.05).

## 3. Results

All 32 specimens, that were artificially aged, passed the cyclic loading without failure and underwent subsequent pull-off tests.

**Figure 8:**
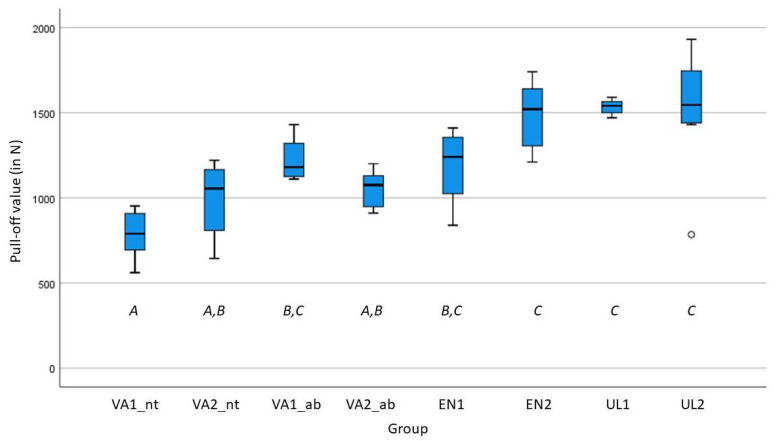
Boxplot diagram showing the pull-off force values [N] of all test groups and their group distribution (*A*–*C*).

The mean pull-off forces were 786.6 ± 137.6 N (VA1_nt), 988.6 ± 212.1 N (VA2_nt), 1223.8 ± 119.2 N (VA1_ab), 1051.9 ± 107.2 N (VA2_ab), 1185.9 ± 211.8 N (EN1), 1485.0 ± 198.2 N (EN2), 1533.8 ± 42.4 N (UL1), and 1521.8 ± 343.4 N (UL2). All test groups showed normal distribution regarding pull-off forces. The visualization of the results of the test groups on the crown pull-off forces was performed using a box and whisker plot (Figure 8). ANOVA followed by the Scheffè method post hoc test revealed the following groups: VA1_nt, VA2_nt, and VA2_ab formed one subgroup, VA2_nt, VA2_ab, EN1, and VA1_ab a second one, and EN1, VA1_ab, EN2, UL2, and UL1 the third subgroup (one-way ANOVA (Scheffé method); *p* < 0.05) (Table 2).

A detailed distribution of the four defined failure modes is given in Table 2.

## 4. Discussion

The first null hypothesis must be rejected since some of the pull-off forces from VA showed significantly lower pull-off values compared to other RMCs. The second null hypothesis can be accepted because an additional pretreatment step of airborne-particle abrasion for VA displayed no significant influence on the pull-off forces.

Considering the pull-off forces of VA, these were significantly lower than EN or UL in three subgroups. The only exception was VA1_ab, which showed average values of 1223.8 N and thus, ranged within the values of EN and UL. Furthermore, looking at the absolute minima of the groups, the forces were 561.0 N for VA, 839.0 N for EN, and 784.0 N for UL (Table 2). Clinically, such high pull-off forces seldom occur. One can further assume, that the application of these high axial pull-off forces might lead to the extraction of a natural tooth rather than to the failure of the adhesive bond. Dietrich et al. indicated forces required for extracting natural teeth to range between 50 N and a maximum of 629 N [16]. Of course, the pull-off force values cannot be transferred one-to-one to clinical practice. Although there are significant differences between the groups in some cases, all pull-off values are in such a high range that these differences are probably irrelevant for clinical long-term application.

As mentioned in the introduction, contrary to these findings and deductions, debonding is a frequent failure mode in the clinical practice of RMC restorations [8,9]. Reasons for this problem might include inadequate moisture control during bonding, inappropriate luting cements, inadequate abutment heights, or too conical preparations [11,17]. Further parameters for successful long-term survival are the skills of the operator and strict adherence to the manufacturers’ instructions, including the required restoration thickness [18,19]. In contrast to the present study, the ideal study conditions cannot be achieved in everyday clinical routine. Furthermore, extra-axial forces occur during normal chewing, which could not completely be considered in an in vitro study. Nevertheless, the experimental set-up was chosen as an attempt to be close to the clinical situation and simultaneously provide a high level of standardization.

The glass fiber-reinforced high-performance polymer (TRINIA, Bicon, Boston) with a Young’s modulus similar to that of dentin or bone around E *=* 18,800 MPa [20] was used to manufacture the tooth stumps #46 (FDI). Due to its mechanical characteristics, the material enables simulating a dentin-like behavior during pull-off tests. This is pivotal, since the Young’s modulus has a significant influence on the retention loss behavior of crowns. Crowns on extracted natural teeth might have been an alternative. However, any standardization and comparability would have been very difficult, since the anatomy varies vastly, which would, in turn, result in large differences in preparation designs, layer thicknesses, morphology as well as enamel and dentin bonding surfaces.

Therefore, the possible critical aspect, that the crown was adhesively luted to a polymer instead of the tooth structure needs to be discussed. Generally, a reliable cohesive bonding between the tooth structure and the luting composite can be assumed [21,22]. In order to achieve an improved adhesion to the TRINIA-tooth stump, the latter was airborne-particle abraded beforehand. Then, the workflow of conventional pretreatment of a tooth surface was performed by Scotchbond Universal adhesive (3M Deutschland GmbH, Neuss). It could be debatable to use Monobond Plus (Ivoclar Vivadent AG, Schaan, Liechtenstein) for this purpose as this is accredited by the manufacturer to be used only on restoration side surfaces.

Further on, adhesive bonding between the restorations and the luting composite seems to be more relevant [23]. Thus, the pretreatment of the restorative material plays a relevant role in this regard. Microretentive surfaces are created either by airborne-particle abrading or etching with hydrofluoric acid, which should subsequently be silanized to ensure chemical adhesion prior to bonding with milled resin composite materials or milled ceramics with polymer infiltration, such as EN [24,25].

EN achieves the best values in terms of surface treatment with 5% hydrofluoric acid etching and should be etched for 60 s if possible, in order to create an optimum microretentive surface on the one hand and at the same time to avoid damage to the ceramic matrix by “over-etching” [26,27]. For UL, Reymus et al. postulated that a successful adhesive bonding of CAD-CAM resin nanocomposites with luting composites requires airborne-particle abrading, as significantly higher tensile strength values can be realized [13]. Findings for VA are not yet available in the literature. In this study, airborne-particle abrasion of the luting surface showed higher adhesion values in absolute numbers, but only partially above the significance level (VA1_ab) and thus, airborne-particle abrasion does not seem to be unconditionally necessary. In turn, it can also be deduced that airborne-particle abrasion does not show negative effects on the adhesion to VA. Especially to avoid possible retention loss in clinical practice, where no idealized in vitro conditions can be assumed, a recommendation of airborne-particle abrasion appears reasonable. Hence, one might suppose that alongside the standard cleaning protocol of polymer crowns in an ultrasonic bath prior to insertion, previous airborne-particle abrasion might remove any possible contamination during the try-in procedure of the restoration.

Material composition of RMCs is a decisive factor for their physical and chemical properties, so that bonding between two materials could also be influenced by this in a manner [28,29]. Furthermore, an influence of residual monomer on bonding is quite possible. After 3D printing, the crowns were additionally subjected to a post-curing process within a light-curing device (BEGO Otoflash, BEGO, Bremen, Germany), which is intended to reduce the residual monomer content. Nevertheless, it can be stated that the probability of residual monomers in 3D-printed objects is higher compared to milled RMCs or resins [30]. However, the results of the present study show that these points seem not to influence clinical applicability. Against the background of the recent market launch of adhesively cemented 3D-printed definitive crowns, no studies are yet available in this regard but should be carried out next.

A uniform failure mode could not be detected in any of the test groups. Complete debondings (#1) between crowns and stumps were identified most rarely. In most cases, crowns were fractured and parts of the stumps were damaged (#2 and #3) (Table 2). This does not reflect the failure modes in clinical practice [8,9]. On the other hand, this observation showed that sufficient adhesive and cohesive tensile strengths can be assumed under ideal conditions. Thus, even if the absolute pull-off values of VA are significantly below EN and UL, a sufficient bonding strength between the stump and the crown can be implied.

It must be clearly mentioned, that multiple steps in the fabrication of the crowns were carried out manually, e.g., removing the connector structures or finishing and polishing the crowns. Furthermore, the cementation procedure in particular is completed manually and therefore underlies human influence. Much effort has been made to minimize these factors by a “standardized” manufacturing process carried out by one trained researcher. Nevertheless, some variances regarding the pull-off values can occur across all groups.

In the present study, the effect of artificial aging on adhesive bonding, represented by pull-off values, should be compared before and after in vitro artificial aging. No failures occurred during artificial aging. In this study, artificial aging did not significantly change the pull-off values as demonstrated by the lack of statistical significance to the before and after pull-off values for all material groups. The pull-off tests of VA2_ab and EN2 revealed pull-off values tending to be higher following artificial aging, even if there was no significant difference between VA1_ab and EN1 (Table 2).

There is no standardized aging protocol for pull-off tests [31], but an aging applying 50 N over 1,200,000 cycles (1.2 Hz) with 10,000 thermocycles (5 °C/55 °C) simultaneously is one common aging protocol for luted crowns [32,33] and simulates a 5-year in vivo aging [34]. However, a comparison of the results of the pull-off tests to other studies does not seem reasonable due to the differences in experimental setups [31].

Retention after adhesive bonding is one of multiple factors which indicate the long-term stability of a restoration. The results of this study suggest a retention after adhesive bonding of VA which is comparable to EN or UL. Nevertheless, further in vitro and clinical studies are needed to verify the results, evaluate further factors affecting long-term stability, and compare among themselves; such as, resistance to extra-axial forces, abrasive behavior, biocompatibility, elution, or color stability over time.

## 5. Conclusions

Within the limitations of this in vitro study, it can be concluded that:

1. Artificial aging does not seem to influence the pull-off forces for adhesively luted crowns of VA, EN, and UL.
2. Uniform failure modes could not be detected in any of the test groups. Nevertheless, pull-off force values determined for all crown materials were within a high range and on comparable, probably clinically acceptable levels.
3. Airborne-particle abrasion of the intaglio surfaces of the crowns made from VA did not result in significantly higher adhesion values. However, it is recommended to ensure the best possible conditions for bonding.

## Figures and Tables

**Table 1:** Overview of tested materials and their group-specific luting protocols (Al_2_O_3_: airborne-particle abraded with aluminum oxide; HF: etched with hydrofluoric acid). ^a^ data from the manufacturers.

Code	Crown Material	Material Type (Monolithic)	Process	Manufacture	Treatment of Restorative Material	Artificial Aging	Flexural Strength [MPa]
VA1_ab	VarseoSmile Crown ^plus^	3D-printed hybrid composite	Additive (3D-printed)	BEGO, Bremen, Germany	50 μm, Al_2_O_3_, 1.5 bar, Monobond Plus	No	116 ^a^
VA2_ab	VarseoSmile Crown ^plus^	3D-printed hybrid composite	Additive (3D-printed)	BEGO, Bremen, Germany	50 μm, Al_2_O_3_, 1.5 bar, Monobond Plus	Yes	116 ^a^
VA1_nt	VarseoSmile Crown ^plus^	3D-printed hybrid composite	Additive (3D-printed)	BEGO, Bremen, Germany	Monobond Plus	No	116 ^a^
VA2_nt	VarseoSmile Crown ^plus^	3D-printed hybrid composite	Additive (3D-printed)	BEGO, Bremen, Germany	Monobond Plus	Yes	116 ^a^
EN1	Enamic	Hybrid ceramic: polymer-infiltrated ceramic networks (PICN)	Subtractive (milled)	VITA Zahnfabrik, Bad Säckingen, Germany, Germany	60 s HF, Espe Sil	No	150–160 ^a^
EN2	Enamic	Hybrid ceramic: polymer-infiltrated ceramic networks (PICN)	Subtractive (milled)	VITA Zahnfabrik, Bad Säckingen, Germany, Germany	60 s HF, Espe Sil	Yes	150–160 ^a^
UL1	Lava Ultimate	Resin nano ceramic or resin-based composite	Subtractive (milled)	3M Deutschland GmbH, Neuss, Germany	50 μm, Al2O3, 1.5 bar, Scotchbond Universal Adhesive	No	191 [15]
UL2	Lava Ultimate	Resin nano ceramic or resin-based composite	Subtractive (milled)	3M Deutschland GmbH, Neuss, Germany	50 μm, Al_2_O_3_, 1.5 bar, Scotchbond Universal Adhesive	Yes	191 [15]

**Table 2:** Overview of group-specific pull-off force values, their statistical classification (group distribution after Scheffè method (*p* < 0.05)), and failure modes; same letters A, B, and C indicate no statistically significant differences concerning the pull-off forces.

Code	VA1_ab	VA2_ab	VA1_nt	VA2_nt	EN1	EN2	UL1	UL2
Pull-off forces								
Arithmetic average [N]	1223.8	1051.9	786.6	988.6	1185.9	1485.0	1533.8	1521.8
Standard deviation [N]	119.2	107.2	137.6	212.1	211.8	198.2	42.4	343.4
Maximum [N]	1430.0	1200.0	952.0	1220.0	1410.0	1740.0	1590.0	1930.0
Minimum [N]	1110.0	910.0	561.0	644.0	839.0	1210.0	1470.0	784.0
ANOVA Scheffè method (post hoc test)								
Subgroup for alpha *=* 0.05	B, C	A, B	A	A, B	B, C	C	C	C
Failure mode								
#1	0	0	4	0	1	0	0	0
#2	4	5	4	5	4	2	0	1
#3	1	3	0	3	0	0	4	1
#4	3	0	0	0	3	6	4	6

## Data Availability

Not applicable.
